# The impact of rainfall on drinking water quality in Antananarivo, Madagascar

**DOI:** 10.1371/journal.pone.0218698

**Published:** 2020-06-15

**Authors:** Alexandra Bastaraud, Emeline Perthame, Jean-Marius Rakotondramanga, Jackson Mahazosaotra, Noro Ravaonindrina, Ronan Jambou

**Affiliations:** 1 Food Hygiene and Environment Laboratory, Institut Pasteur of Madagascar, Antananarivo, Madagascar; 2 The Center of Bioinformatics, Biostatistics and Integrative Biology (C3BI), Institut Pasteur Paris, Paris, France; 3 Epidemiology Department Institut Pasteur of Madagascar, Antananarivo, Madagascar; 4 Global Health Department, Institut Pasteur Paris, Paris, France; University of Minnesota Twin Cities, UNITED STATES

## Abstract

Low-income cities that are subject to high population pressure and vulnerable to climate events often have a low capacity to continuously deliver safe drinking water. Here we reported the results of a 32-year survey on the temporal dynamics of drinking water quality indicators in the city of Antananarivo. We analyzed the long-term evolution of the quality of the water supplied and characterized the interactions between climatic conditions and the full-scale water supply system. A total of 25,467 water samples were collected every week at different points in the supplied drinking water system. Samples were analyzed for total coliforms (TC), *Escherichia coli* (EC), intestinal Enterococci (IE), and Spores of Sulphite-Reducing Clostridia (SSRC). Nine-hundred-eighty-one samples that were identified as positive for one or more indicators were unevenly distributed over time. The breakpoint method identified four periods when the time series displayed changes in the level and profile of contamination (i) and the monthly pattern of contamination (ii), with more direct effects of rainfall on the quality of supplied drinking water. The modeling showed significantly different lags among indicators of bacteria occurrence after cumulative rainfall, which range from 4 to 8 weeks. Among the effects of low-income urbanization, a rapid demographic transition and the degradation of urban watersheds have gradually affected the quality of the water supplied and resulted in the more direct effects of rainfall events. We focused on the need to adopt an alternative perspective of drinking water and urban watersheds management.

## Introduction

A poor capacity to provide safe drinking water, regardless of weather conditions, is of growing concern in low-income areas vulnerable to climate change [1,2]. Indeed, some parts of the world are expected to experience an increase in the frequency and intensity of precipitation and will find it increasingly difficult to limit the impact of storms [3], such as flooding or heavy run-off [4,5]. These events are associated with elevated turbidity [6–8] and dissolved organic matter in water sources [9], which can overwhelm treatment plans [10]. The high levels of suspended solids limit the proper progress of the clarification steps and reduce the efficiency of chlorination. Indeed, extreme rainfall regimes are likely to be associated with drinking water contaminations [6,10], and this is predicted to be worsened by climate change [11]. Contaminated water is the main cause of diarrhea in children, and it is evident that an integrated approach to improving water supply will have an impact on the health of the population [12].

However, the relationship between rainfall patterns and microbial water quality is complex, involving an interplay between the type of water supply, the type of water source, and the treatment technology applied to water [13]. Also, susceptibility to climate change is reinforced by rapid and unplanned urbanization, poor sanitation, erosion, and low level of maintenance of the supply network [14]. Thus, the nature and the depth of the link between rainfall and water quality is not expected to be stationary. Rather, these should vary with the infrastructure and environmental changes, the time scale of study (yearly, season or day to day), and the rainfall patterns. Rainfall is a seasonal phenomenon with significant inter-annual variability [15] related to climate variations [16], extreme climatic events [6], intra-annual variability or distribution of water [17], change in duration of spells of continuous rain or no-rain events, and the total amount of water delivered during each wet spell [15]. Combined or selective impacts of these factors also depend on the catchment’s characteristics.

Due to its diverse landscape, Madagascar is exposed to a variety of weather and climate phenomena [18]. The capital of Madagascar (Commune Urbaine d'Antananarivo–CUA) has experienced rapid urbanization due to the arrival of an additional 100,000 inhabitants per year. This population growth has increased the technical constraints on infrastructure and local services that were already deficient [19]. Water supply in Madagascar dates only from the colonial period (1952) and has been outpaced by urban expansion and population growth. Consequently, home connections are still limited, and a network of about 900 standpipes (public water fountains) supplies un-piped households. In this context, storm events, heavy rainfall, and runoff can increase water turbidity and microbiological contamination [20]. Un- or insufficiently filtered, inadequately, and even adequately disinfected drinking water is thus susceptible to microbiological contamination [21]. Water utilities are required to monitor microbial indicators to assess the effectiveness of the treatment process (i.e., spores of *Clostridia*), the safety of end drinking water (i.e., *Escherichia coli*, intestinal enterococci), and the biological stability of microorganism communities in piped water (i.e., total coliforms) [21]. All changes impacting the water source or the treatment process can affect these indicators and the drinking water quality. Exploring the interaction between precipitation events and urban water supply systems will highlight monitoring needs and priorities for improving water production.

Due to its geographic setting, its demographic burden, and its environmental transition, Antananarivo is a suitable example for setting up new strategies to survey water treatment based on predictive mathematic models. Predictive models could also be used to adapt the activities of dispensaries based on the burden of diarrhea. First, we analyzed how the water quality had evolved over the last 30 years, and then how the rainfall pattern has impacted long-term water quality in the supply system under environmental and technical shifts. We also focused on the current week-to-week relationship between rainfall patterns characteristics and water quality to determine the main factors regulating water contamination.

## Materials and methods

### CUA and its drinking-water supply system

#### Site of study

**Fig 1:**
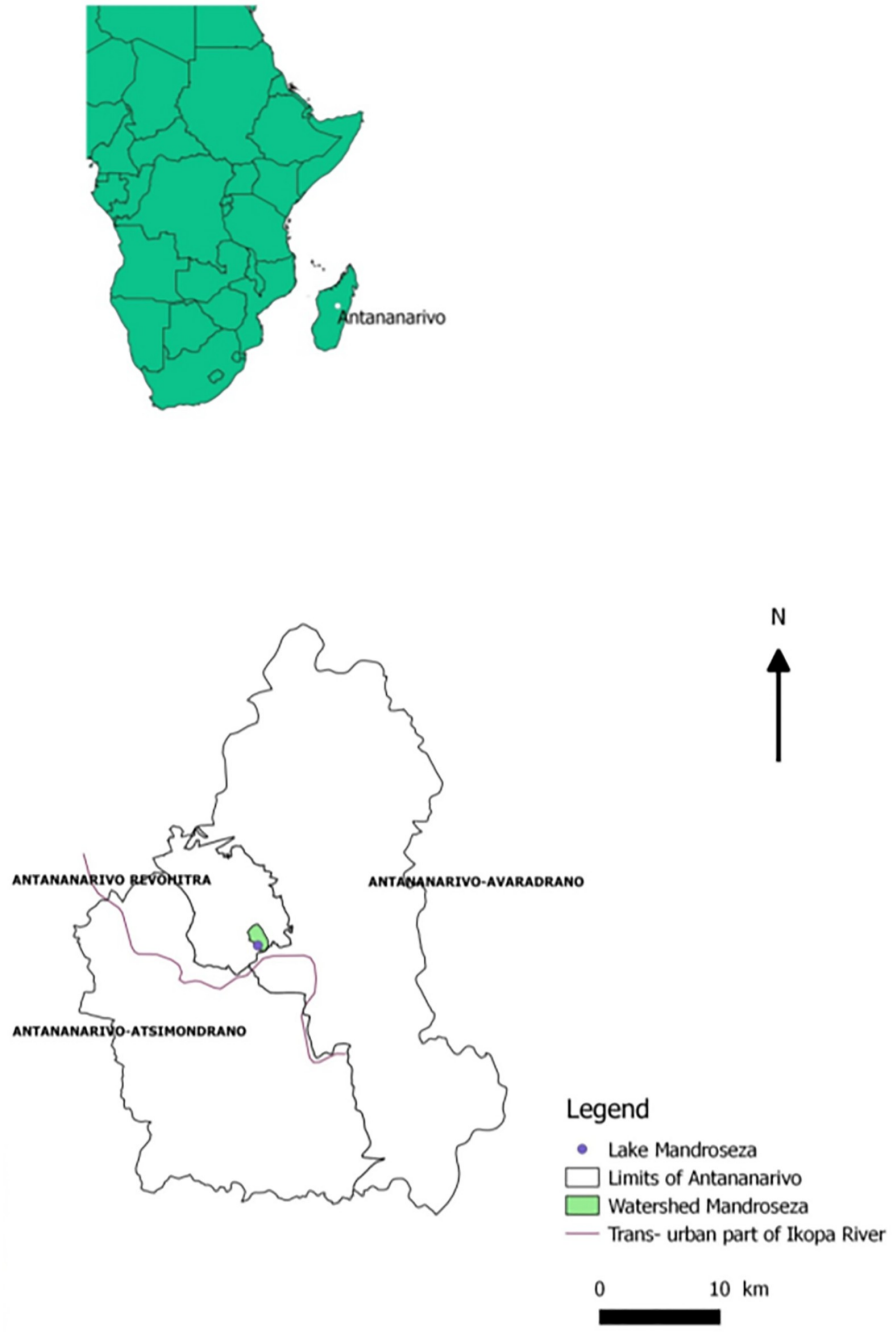
Location of Mandroseza Lake inside the limits of Antananarivo. The file in “Africa map” created from OpenStreetMap databank are licensed under the Open Database 1.0 License. Administrative boundaries were from data OCHA (office for the coordination of Humanitarian Affairs) https://data.humadata.org/dadaset/madagascar-administrative-level-0-4-boundaries. Watercourse layers are OpenSource databases from https://www.openstreetmap.org. OpenStreetMap ^R^ is *open data*, licensed under the Open Data Commons Open Database License (ODbl) by the OpenStreetMap Foundation (OSMF). The final map was prepared with own tiles under “ARCGIS software”.

Antananarivo is located in the central highlands of Madagascar at 1,300 m above sea level (18°55’ S latitude and 47°32' Longitude). The city is nestled among twelve hills and lies in the natural floodplain of the Ikopa River, which skirts the city to the south and west (Fig 1). The river and its tributaries play an important role in rice-dominated agricultural production. The metropolitan area spreads over 220 km^2^, with an estimated population of 3,058 million inhabitants in 2018 [22]. This area currently experiences significant challenges due to flooding during the rainy season.

#### Climate

Antananarivo experiences a subtropical highland climate, warm and temperate classified as Cwb by the Köppen-Geiger system [23]. Summers are rainy, with most of the precipitations falling in December, January, and February. The winters are dry, especially in June, July, and August. The dry season occurs from May to September (i.e., from week no.14 to no.40), and the wet season from November to April (i.e., from week no.41 to no.13). From 1985 to 2017, the annual average of rainfall was above 1500 mm with extremes in January (above 300 mm) and June (less than 10 mm). The city of Antananarivo has experienced cyclones over the past 20 years, including Geralda in January 1994, Giovanna in February 2012, and Enawo in January 2017. These induced severe flooding as in February 2015 and 2016.

#### Water supply

In 2015, according to the National Water and Electricity Utility (JIRAMA), the whole drinking water supply system represents 1000 km of pipes for 80,000 subscribers (supply rate 56.8%). Water is provided by the Ikopa River, whose flow is diverted to the 1.41 km^3^ artificial Mandroseza Lake from where water is pumped [24]. Two water stations, Mandroseza I and II, with a daily production of 93,000 and 62,000 m^3^ per day (m^3^/d) respectively, supply 30 reservoirs. The treatment process includes coagulation, flocculation, decantation, filtration, chlorination, and neutralization steps [25].

### Data collection

#### Water sampling and analysis

Four different points in the network (e.g. piped households, administrative buildings, standpipes, water tanks) were randomly investigated daily, five days a week. Each sample was collected in 500 ml sterile containers with 10 mg sodium thiosulfate and stored at 4 to 10°C until processing at the Institute Pasteur laboratory within 18 to 24 hours. Microbial water quality was assessed by enumerating samples contaminated or not by microbial indicators, including *Escherichia coli* (EC) and Total Coliform count (TC); intestinal enterococci (IE); spores of Sulfite-Reducing Clostridia (SSRC) [26]. From 1985 to 2014 (August), the laboratory used standardized methods based on the filtration of 100 milliliters (ml) of sample for testing EC, TC [27,28] and IE [29,30]. Since August 2014, the IDEXX Quanti-Tray methods were performed for testing IE [31], EC and TC [32]. From 1985 to June 2010 and after August 2016, the detection and enumeration of the spores of sulfite-reducing anaerobes (SSRC) required an enrichment of 20 ml of sample in a liquid medium [33]. From July 2010 to July 2016, the 100 ml filtration method was preferred for testing SSRC, resulting in a five-fold increase in test volume [34]. Criteria for a negative sample is set to “undetectable microorganism in any 100 ml” and in any “20 ml for SSRC”.

Temporal patterns of microbial water quality are expressed as the frequency of positive samples reported to the total number of samples collected during the period (monthly and weekly) over 32 years (from 1985 to 2017, except 2009 due to the insurrectional crisis). Monthly series had 6.41% missing values, and weekly series had 18.69% missing values. The missing values were mainly due to the interval between analysis service contracts or weeks during which samples were not taken (technical problems or weeks not working). For multivariate analysis, missing data were replaced by the median of the related month for monthly series and of the related week for weekly series.

#### Rainfall data

From 1985 to 2017, monthly rainfall was collected from the data from *Direction Générale de la Météorologie*. Daily rainfall (available from 2007 to 2017) was collected from the IRI-International Research Institute for climate and society. To have the same time-step for rainfalls and contamination, these data were summarized as cumulative rainfall by month or by week.

### Statistical analysis

#### Breakpoint detection method

To detect specific periods or obvious trends within contamination markers and rainfall time series, we applied a breakpoint detection method implemented in the Strucchange R package [35]. We used the method of simultaneous estimation of multiple breakpoints proposed by Bai and Perron in 2003 [36]. The method was run using the default parameters of breakpoints function, and the number of periods was estimated by minimizing the BIC (Bayesian Information Criterion).

#### Fourier analysis

To test if the variable “month” affects the contamination markers and rainfall pattern, we applied a Fourier transform to each variable using the TSA (Time Series Analysis) R package [37]. The computed periodograms from this transformation were tested. If there is a month effect in the time series, the periodogram should have a peak at time 12 (corresponding to 12 months). The significance of the amplitude of the periodogram at time 12 is tested using a permutation test (i.e., comparison with a random sequence with a significance level at 0.05) [38]. All p-values are available in the supporting information (S1 Table).

#### Hierarchical clustering

To check for similar current profiles of contamination, we focused on data from the last period provided by statistical analysis of contamination change. We applied a hierarchical clustering algorithm with Euclidean distance and Ward distance. The clustering was applied to the four contamination markers (IE, EC, TC, and SSRC). Total contamination was not used for clustering to avoid collinearity with IE, TC, EC, and SSRC. Rainfall and total contamination were added to the graphical representation for interpretation.

#### Auto-regressive integrated moving average (ARIMA) models

To investigate the specific relationship between drinking water contamination and rainfall, we have run three models following: a “naive” model (i) that consists of forecasting the contamination of a given week by the mean of the previous corresponding weeks. This model does not account for the effect of rainfall and is used as a benchmark for further comparisons: any prediction model achieving higher prediction error is not relevant. Two different ARIMA models are fitted on each marker: an ARIMA model, with no extra covariate (ii); and an ARIMA model adjusted on cumulative rainfalls of the previous weeks (iii), with a shift varying from 1 to 10 weeks. The optimal number of cumulative weeks is estimated by minimizing the prediction error (root mean square error, RMSE) assessed by cross-validation in the years 2016 and 2017. A likelihood-ratio test was used to compare the goodness of fit of statistical models. All parameters of ARIMA models are automatically selected using a stepwise procedure which minimizes the BIC, implemented in the auto.arima function of forecast package [39]. The Portmanteau test was used to conclude that no residual autocorrelation remained in the models [40]. This procedure allows investigation of the effect of rainfall and cumulative rainfalls on contamination markers; it also allows for the quantification of the number of cumulative weeks optimal to predict contamination.

## Results

### Contamination of the water over the 32 years

A total of 971 samples have been identified as positive for one or several microbial indicators among 25,467 samples collected over 32 years (365 months). This accounts for 3.8% of non-compliant samples, unequally distributed over time. Indeed, during the period from 1989 to 2004 (175 months = 75% of the months), no contamination was reported.

### Breakpoints in the yearly pattern of contamination

The time series of monthly water contamination frequencies showed significant shifts over the years for all indicators (IE, EC, TC, and SSRC). Change points and the associated 95% confidence intervals for total contamination (IE, EC, TC, and SSRC), as well as rainfalls, are summarized in Table 1 (Breakpoint detection method).

**Table 1:** Breakpoints in contamination markers and rainfall time series.

	TOTAL ^d^	IE ^c^	EC	TC	SSRC	Rainfall
Breakpoint n°1	1989(1)^a^ [1989(9);1993(4)] ^b^	-	-	1990(1) [1989(12);1991(7)]	-	-
Breakpoint n°2	2004(11) [2003(6);2005(3)]	-	2003(11) [1994(10);2004(9)]	2004(11) [2002(10);2004(12)]	-	-
Breakpoint n°3	2012(3) [2009(10);2012(6)]	2012(8) [2007(6);2014(2)]	-	-	2011(7) [2009(11);2011(9)]	-

^a^ Year and (month) when a breakpoint has occurred;

^b^ 95% Confidence intervals of time when a breakpoint has occurred;

^c^ Contamination markers, namely intestinal enterococci (IE), *Escherichia coli* (EC), total coliforms (TC) and spores of sulfite-reducing clostridia (SSRC);

^d^ Total contamination, regardless of markers.

**Fig 2:**
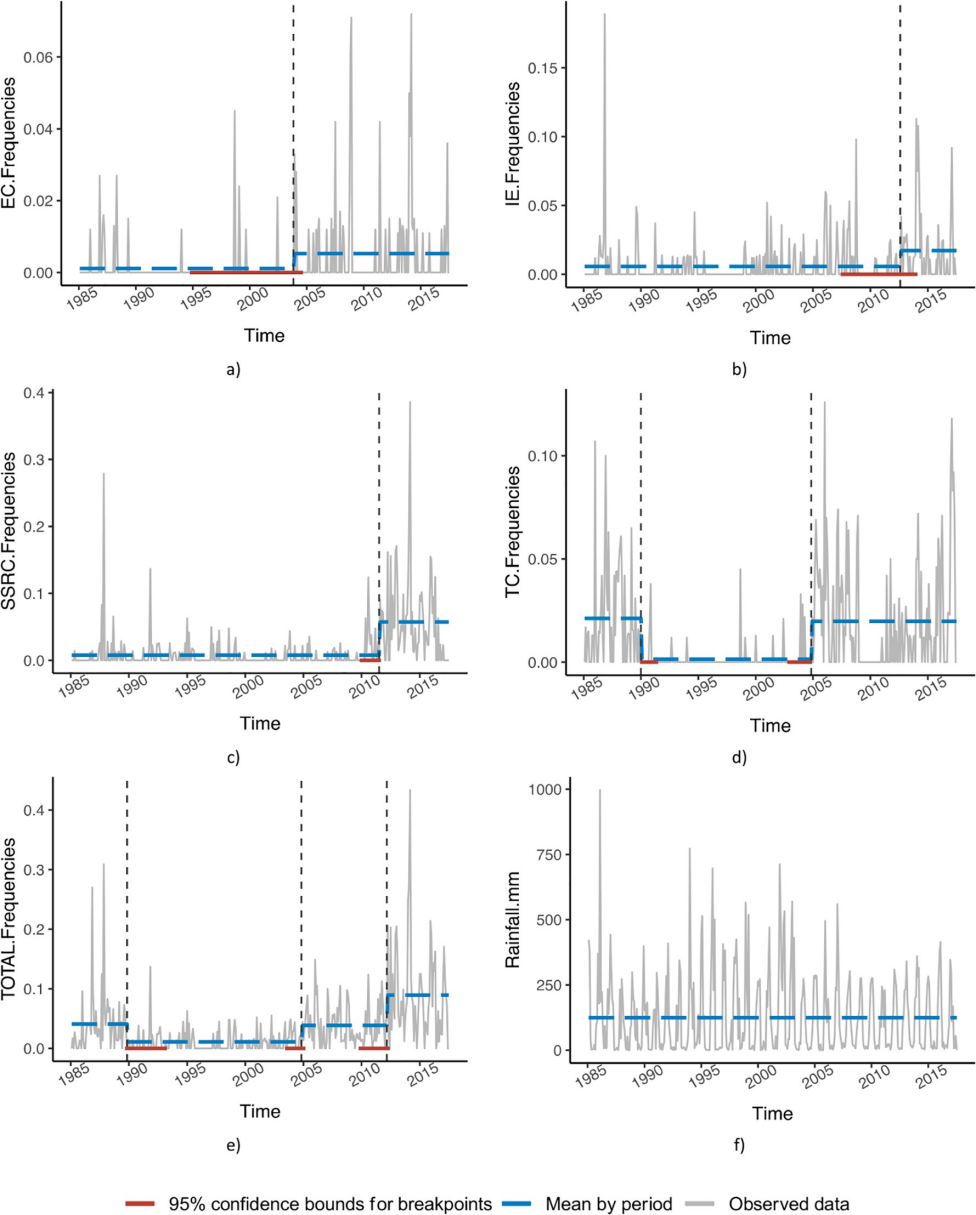
Time series of drinking water contamination frequencies in Antananarivo’s (Madagascar) water supply and rainfall from 1985 to 2017, using the period from breakpoints method. The time series are displayed in grey. The periods are represented by dashed vertical black lines; the mean of the time series within each period is indicated by a dashed blue line. Confidence intervals associated with change points are shown as red lines.

Three breakpoints occurred over the “total contamination” series, defining four periods: i) before 1990, ii) between 1990 and 2005, iii) between 2005 and 2012, and iv) after 2012. Time series analysis also captured specific change-points for each specific contamination marker [i.e., 1990 and 2004 (TC); 2003 (EC); 2011 (SSRC) and 2012 (IE)]. For these periods, the monthly average of contaminated samples varied from 1.1% (the intermediate period from 1990 to 2005) to 9.5% (the last 5 years) (Fig 2). Periods 1 (from 1985 to 1990) and 3 (from 2005 to 2012) showed similar levels of contamination (4.7% and 4.2%). While the period from 1990 to 2005 was relatively free-from water contamination. The period from 2012 to 2017 showed a significant decrease in drinking water quality. Over the 30 years, no breakpoint or obvious trend was detected in the rainfall.

**Fig 3:**
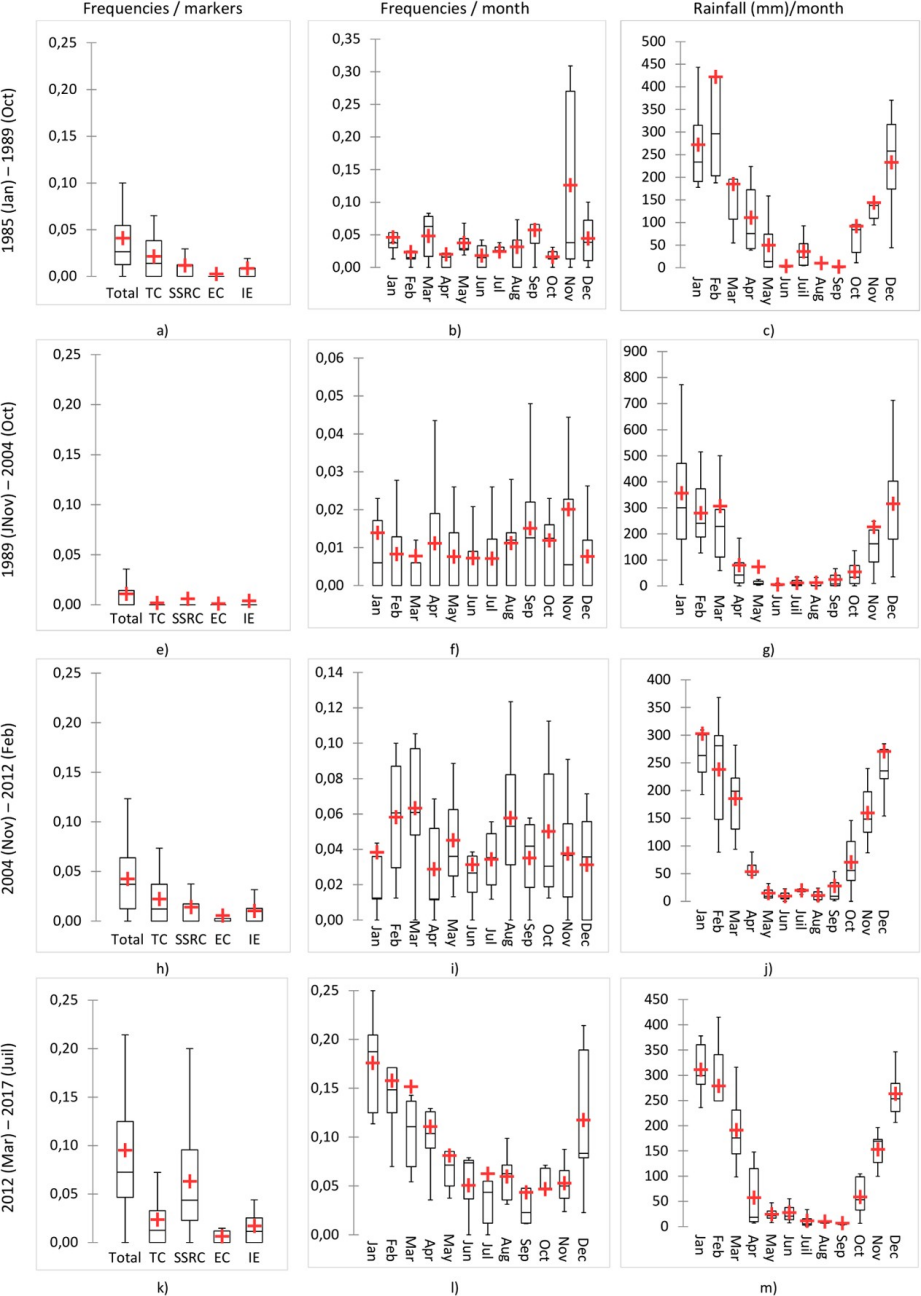
Distribution of drinking water contamination frequencies in Antananarivo’s (Madagascar) water supply and rainfall from 1985 to 2017. Box plots are displayed with a mean (red cross).

Over the whole series, and except for the 1990–2005 period, SSRC contamination events have continuously increased (Fig 3d), with the recent median of contamination events reaching 4.8% (Fig 3k). During the last period, IE contamination also increased, with median contamination events rising from 0 to 0.8% (Fig 3k). EC contaminated samples remained sporadic throughout the periods, with medians of the periods close to zero. However, the means of contamination increased very slightly from 0.1% (Fig 3e) to 0.7% (Fig 3k) (breakpoint in 2004). For TC, three out of four periods showed baseline contamination events with half of the months harboring 1% of contamination.

### Change in monthly pattern of contamination

As the months harboring the highest contamination events also varied over time (Fig 3), the month effect had to be tested independently from the periods. The periodogram method and permutation test showed significant 12 months periodicity in data of several years for TC, IE, and rainfall (p-value < 0.05) (S1 Table). This means that TC and IE contamination events and rainfalls have preferentially occurred at specific months during the year. For SSRC, the test was barely significant (p-value ~ 0.05), suggesting that it suffers from a lack of power to detect a specific pattern for this parameter. No month effect was detected for EC (p-value ~ 0.3 > 0.05).

From 1985 to 2004, November harbored the maximum of sample contamination accounting for the highest mean of the period, with 12.6% (Fig 3b) and 2% (Fig 3f), in the first and second period, respectively. In Antananarivo, November is also the month of the first heavy rainfall (Fig 3c, 3g, 3j and 3m). During the following periods, contamination events progressively increased preferentially at the beginning of the year, with means and medians of contaminated samples reaching 6% and 17% respectively in March (third period) and January (last period) (Fig 3i and 3l). This is in accordance with the rainiest months (Fig 3j and 3m). During the last 6 years, contamination events spread over the December to April period (Fig 3l). Thus, monthly contamination distribution can be superposed to rainfall distribution (Fig 3l and 3m).

### Relationship between water contamination and rainfall

#### Profile contamination clustering

**Fig 4:**
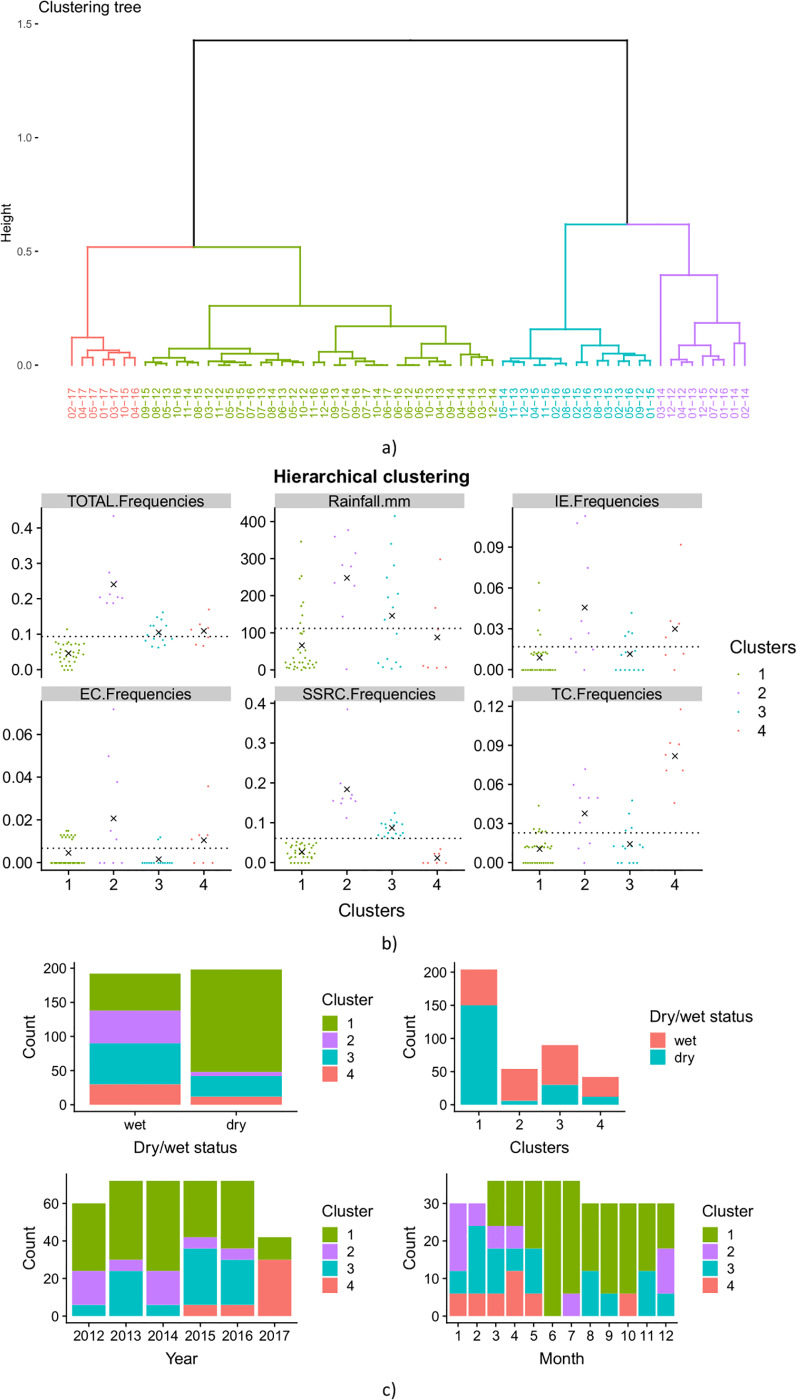
Hierarchical clustering of monthly observations from drinking water monitoring in Antananarivo’s (Madagascar) water supply from 2012 to 2017. a) Clustering tree; b) Scatter plot displays the distribution of all markers within each cluster. The black crosses are the mean within the cluster of the corresponding variable. The dotted black line is the overall mean. c) Bar plots explore the repartition of each cluster by wet/dry seasons, by year and by months.

Correlation between the percentage of contaminated samples collected for one month and the rainfall measured during the same month was first queried using a clustering strategy conducted on the whole data set collected from March 2012 to the current date. For each month, rainfall, total contamination, IE, EC, TC, and SSRC were considered as variables for multiple component analysis (MCA) and automatic ascendant classification. The months grouped in a cluster and exhibited a similar profile of contamination. Fig 4a displays the clustering tree. Four clusters were determined according to the level of each contamination marker (IE, EC, TC, and SSRC). The scatter plot displays the distribution of all markers within each cluster. Rainfall and total contamination were also displayed (Fig 4b). Cluster 1, the largest, included 34 observations characterizing to months with low contamination in the context of low rainfall. Cluster 4, the smallest, included seven observations that demonstrated high contamination in TC and IE/EC, in the context of middle rainfall level. Clusters 4 and 3 exhibit similar total contamination. However, cluster 3 reported higher SSRC, lower TC, and IE/EC contamination and higher rainfall than cluster 4. This suggests that a similar total contamination rate might be associated with different contamination profiles. Cluster 2 demonstrated high contamination for all markers in the context of high rainfall.

In summary, during the dry seasons, the level of contamination was relatively low and mainly caused by SSRC (cluster 3). During the wet seasons, contamination was related to all other contamination markers (cluster 2) or by SSRC (cluster 3). Contamination by TC and IE/EC (cluster 4) was also more reported during the wet season.

The contamination profile also varied over the years. TC and EI/EC contaminations emerged three years ago and became predominant in 2017 (cluster no.4). Whereas in 2013, 2015, and 2016, contamination was mainly sustained by SSRC (cluster no. 3) and in 2012 and 2014 by all other markers (cluster no. 2). At the month level, contamination profiles varied cyclically according to the seasons. The wettest months of January and February showed high contamination, mainly caused by all types of microorganism (cluster no. 2) and SSRCs (cluster no. 3) respectively. This high contamination level occurred until May, regardless of the marker. Except during these first 5 months of the years, low or no contamination was observed, suggesting a rainfalls effect on emergence and persistence of contamination.

#### Rainfall and contamination modeling

**Fig 5:**
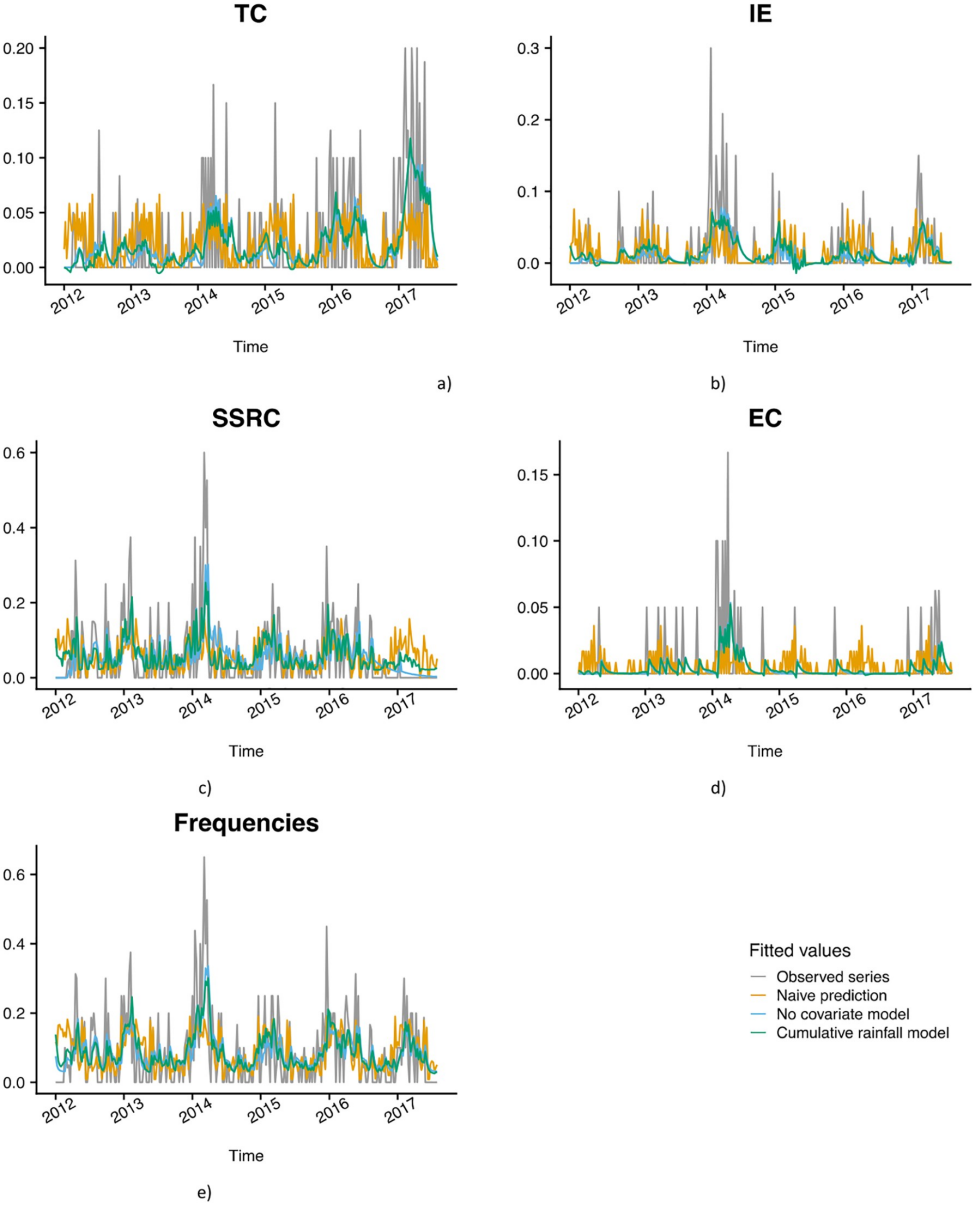
Modeling drinking water contamination in Antananarivo’s (Madagascar) water supply from 2012 to 2017. The following figure shows the observed series in grey, the values fitted by the mean in orange, by the ARIMA model with no covariate in blue, and by the ARIMA model adjusted on the optimal number of cumulative rainfalls in green.

According to previous data, the cumulative effect of the amount of rain fallen over previous weeks on contamination levels can be suspected. Three models (observed means, ARIMA model with no covariate, and ARIMA model adjusted to the optimal number of cumulative precipitations) were compared to select the one that best predicted the impact of cumulative rain on the occurrence of each contamination marker. The values fitted by the three models were reported (Fig 5), and determinants of best models were summarized in Table 2.

**Table 2:** Determinants of best ARIMA model adjusted to the optimal number of cumulative rainfalls.

Contamination markers^a^	Lag (weeks)	BIC ^b^ model	BIC no-covariate	Likelihood ratio^c^	Prediction model^d^	Prediction naïve	Prediction no-covariate
IE	5	-1119.84	-1118.34	7.4E-4	3.26E-2	3.62E-2	3.38E-2
EC	-	-1460.62	-1465.96	5.6E-1	1.44E-2	1.87E-2	1.46E-2
TC	8	-1010.19	-1011.11	2.95E-2	6.07E-2	6.07E-2	6.26E-2
SSRC	4	-620.49	-608.22	2.0E-5	6.26E-2	8.55E-2	6.61E-2
Total	5	-546.67	-542.62	1.9E-3	7.95E-2	8.91E-2	8.65E-2

^a^ Contamination markers, namely intestinal enterococci (IE), *Escherichia coli* (EC), total coliforms (TC) and spores of sulfite-reducing clostridia (SSRC);

^b^ Bayesian Information Criterion values for adjusted and no-covariate (reference) models (the lower, the better);

^c^ Likelihood ratio to test if the adjusted model is a better fit than the naïve model (values must be <0.05);

^d^ Prediction accuracy of the three models (the lower, the better): adjusted model over cumulative rainfall weeks (i), naïve model based on previously observed means (ii) and model with no-covariate.

The ARIMA model adjusted with cumulative weekly rainfall was found as the most accurate with the lowest BIC value and with a significant ratio test of likelihood (p-value <0.05). The Portmanteau test concludes that no residual autocorrelation remained in the models (p > 0.05 for all models).

In summary, apart from EC, these models showed that weekly cumulative rainfalls had an impact on drinking water quality with different time lags according to the contamination markers. For total contamination, a lag of 5 weeks of cumulative rainfall led to the best model (BIC = -546.67) when comparing to the model with no covariate (BIC = -542.69, a p-value of the likelihood ratio test = 1.9e-03<0.05). Prediction performance is also better than the other models (7.95E-2 < 8.91E-2 and 8.65E-2).

For other specific markers and in agreement with MCA, the different lags suggest the chronological emergence of contamination markers after the weekly rainy periods: 1) SSRC contamination events generally occurred first after 4 weeks of cumulative rainfalls; 2) IE contamination events occurred after 5 weeks of cumulative rainfalls; 3) TC emerged last, within 8 weeks of cumulative rainfalls. For EC, this procedure estimated that 3 weeks was the optimal cumulative rainfall (BIC = -1460.62). Nevertheless, the likelihood of the model does not significantly increase compared to the model with no covariate (BIC = -1465.96, a p-value of likelihood ratio test = 5.6e-01 > 0.05). This suggests that the procedure is not able to detect how rainfall affects EC rate. Prediction error of the model adjusted on 3 weeks of cumulative rainfall (~ 1.44e-02) does not lead to an improvement of the prediction accuracy of the naive model (~ 1.87e-02) nor of the ARIMA model with no covariate (~ 1.46e-02). This could be due to a lack of power of the model as EC contamination events were too sporadic (9% of the series).

In conclusion, the emergence of SSRC, IE, and TC are differentially linked to cumulative weekly precipitations, but no significant impact of cumulative rainfall could be detected for EC.

## Discussion

### Drinking water quality issues

Thirty-two-year monitoring of microbial indicators was performed in Antananarivo's full-scale drinking water distribution system, which operated under severe pressure from rapid and unplanned urbanization. The study showed that seasonal variations and significant long-term changes occurred in the microbiological quality of drinking water in the Antananarivo supply system. Large variations were observed in the occurrence of intestinal enterococci—EI (indicator of fecal pollution), total coliforms—TC (indicator of treatment efficiency or cleanliness and integrity of distribution systems) and clostridia spores—SSRC (indicator of filtration plant performance), following rainfall. Such seasonal variations in drinking water system performance were potentially due to rapid changes in raw water quality as a result of precipitation (e.g. increased stormwater flows and discharges, soil erosion, sporadic high turbidity) and an increased microbial load entering drinking water distribution [41] (e.g. overloading of the treatment process, adversely affecting disinfection efficiency) [17,42,43].

Long-term changes were also observed in the annual contamination level of TC, IE and SSRC. Such breakpoints (1993, 2004 and 2012) were potentially attributable to the treatment plant upgrades (Mandroseza II, in 1993, with an increase of 60,000 m3 of water per day), a gradual inability to meet quantity requirements since 2004, and environmental changes that gradually led to rapid and significant fluctuations in raw water quality (e.g. changes in land use, a deforested and urbanized watershed) [43–46]. On the other hand, the low proportion of EC-contaminated samples has shown that, regardless of the ecological context or the technical performance of the treatment plan, the process has always been able to remove recent faecal contamination [47].

### Water supply and demand issues: Signal of imbalance

Over the past 30 years, the capacity for drinking water treatment has not fully met the growing needs of residents. The population in 2017 was 2,904,000, a 5% increase per year from 1985. At the same time, daily water production increased by only 100,000 to 160,000 m3. In addition, the drinking water infrastructure was ageing and falling apart, with a production efficiency of around 60% [22,25,48].

Most pumps, basins, sand filters, storage reservoirs and underground water pipes were installed 60 years ago. Even with this system still in operation, it would need to be upgraded to meet exponential water demand (as an increase of 2% per year) [48]. The situation (e.g. age and no innovative design) required finding a balance that has reconciled duration and efficiency of treatment (nominal capacity of treatment plant) with daily water demand. However, the increase of technical problems, the decrease of yields, and the high seasonal changes in raw water did not allow a long-time balance to be maintained. Water demands in permanent excess of the nominal capacity resulted in a baseline remaining contamination by coliforms that characterized the water supply system.

The installation of the Mandroseza II treatment unit has led to a significant improvement in the microbiological quality of the water (statistical analysis of breakpoints). In practice, the implementation of the filtration step with the double-layer filter in complement of sand filtration has led to a sustainable reduction (more than 10 years) of contamination events in the supply system, especially samples contaminated by TC. Efficiency of clarification step was able to reduce TC contamination by a factor of three [49]. In 2004, the number of contamination events rose again to reach 4% of contaminated samples per month. The TC parameter governed this increase (+3%). The imbalance of the production system therefore shaped the CT contamination level of the water supply system [24,50].

In March 2017, a new subunit (Mandroseza II bis) was implemented to increase the capacity from 3,000 m^3^/h to 3,900 m^3^/h, but it was too soon to assess the impact of this on water quality. However, Antananarivo is expected to host nearly 3,400,000 inhabitants in 2020. Urbanization and demographics can again affect the balance between water supply and demand. The critical point will be the economic capacity to again upgrade infrastructures and to find a new economic model for water supply [51].

### Ecological disruptions and damage to the water resource

Although there were no clear trends in the precipitation data, Antananarivo (since 2012) experienced successive extreme weather events, which led to episodes of high contamination during the first months of the year [52]. Cyclone Giovanna in February 2012 coincided with the start of the period with the highest contamination rates. The rains brought by cyclone Felleng in January 2013 raised river levels, with damage on water infrastructures. The rains of February 2015 triggered floods and the rising floodwaters have broken through several dams around the capital. Heavy rains that have hit the island since late 2014 were followed by Cyclone Chedza in January 2015. Since December 2015—and more precisely in January 2016—Antananarivo has also experienced torrential rains which caused significant damage. Enawo (a category-four tropical cyclone), hit Antananarivo in March 2017 and caused severe floods and landslides.

Significant associations between increased precipitation and greater occurrence of bacterial indicators in water samples were found, with specific lags in the effects of precipitation according to the different indicators. The emergence of SSRC and IE generally occurred within 4–5 weeks of rain, while TC appeared after 8 successive weeks of precipitation. The 4–8 weeks lag effect we observed can be explained by a cumulative phenomenon or a chain reaction that began with rainy season and that affected treatment efficiency, then the hygiene of supply network.

The emergence of the SSRC first was potentially attributed to soil leaching [53] during the first rains in November, which gradually overloaded the station with suspended matter, after 4 weeks. The Ikopa River watershed was severely impacted by erosion (e.g. deforestation, soils poorly protected by vegetation, agricultural practices) [54–57], that adversely affected clarification step and efficiency of disinfection [43]. Highlands cities that used surface water had contamination events, mainly sustained by the SSRC [58]. Failures in the treatment system appeared to have occurred, especially during wet periods.

The first IE emerged at week 5 after the beginning of heavy rainfalls. Their presence was potentially due to a loss of efficiency of filtration systems and chlorination steps (turbid water should be fully clarified to enable disinfection to be effective) and to a greater charge of suspended solids in raw water [59,60]. This increase in IE was only seen during the more recent period. Indeed, in 1995, the silting of the Ikopa River was estimated at 81 m^3^ per year per km^2^ of the watershed, and sediments concentrated mainly upstream of dams (particularly Mandroseza dam) [9,61–63]. Since then, the depth of Lake Mandroseza had gradually decreased from 7.5 m to 3 m and has begun to be invaded by non-aquatic plants.

Demographic and ecological changes have also occurred, including deforestation of watersheds and disturbance of the protection perimeter related to urbanization. The Mandroseza basin has increased from 30 to 50 hectares of the urbanized area [64]. Since then, although EC contamination events are not significant (median zero), the range of contaminated samples has increased. Nevertheless, the emergence of EC contamination is not significantly related to cumulative rainy weeks. The treatment system was able to limit the occurrence of EC, even though urban runoff could be heavily loaded with this bacterium [65].

After 8 weeks of cumulative rainfall (January–February), TC appeared. These contamination events were delayed and not directly related to precipitation. Unlike SSRC and IE, these contamination events seem related to another parameter. This event could be the accumulation of sediments or the deterioration of the cleanliness of the supply networks. The loss of filtration efficiency also creates conditions for the proliferation of TC in the supply network [21,66].

### Bias induced by changes in monitoring

Over the last 30 years, some parameters have been ignored during water quality monitoring. Sample turbidity measurements, for example, have only been recorded since 2016. Similarly, the evaluation of organic matter in the water network (the simplest being the determination of permanganate oxidation) would also be necessary to assess hygiene. Monitoring of the chlorine level should allow the characterization of the response to this phenomenon. The chlorine demand resulting from the difference between the amount of chlorine added and the residual chlorine in the system tends to increase as the hygiene conditions of the system deteriorate. It is also likely that the disruption in 2012, which was characterized by a high level in SSRC, was related to the change in method and volume (× 5) for the measurement of SSRC (NF T 90–415 vs. NF EN 26461–2). Apart from this case, no testing changes impacted on fecal contamination (EC and IE) and TC, which were highly stable during this period.

## Conclusions

The bacteriological quality of the supplied water in Antananarivo has gradually deteriorated in recent years. Water supply infrastructure did not kept pace with population growth and the imbalance between production capacity and water demand has become critical (exponential urban growth and low production efficiency), with a serious impact on the quality of supplied water.

Unplanned urban expansion and land-cover change (deforested watershed) reinforced the impact of heavy rainfall on drinking water quality (high variation of suspended solids). Siltation of lake resources and erosion were aggravating factors during rainy periods, introducing contamination markers attached to sediments into the supply system (i.e., spores of sulfite-reducing of Clostridia and intestinal enterococci).

The overload of the filtration system mainly occurred after four weeks of cumulative rainfall favoring strong contamination in January and February. Regrowth conditions of bacteria were evident with the emergence of total coliforms after 8 weeks of cumulative rainfall. Consequently, the vulnerability to persistent contamination and biological instability generally persisted during rainy periods. On contrast, Escherichia coli were generally removed by the implemented treatment, even during periods of heavy rainfall.

The upgrading of the treatment plant in 1993 had a long and positive impact on drinking water quality, mainly in decreasing contamination events by total coliforms. Appropriate upgrading of the filtration process could be effective in improving the microbiological quality of the water in the supply system. Otherwise, a fair balance between the duration of filtration (flow rate) and the quantity of available treated water must be found.

Stability in testing methods and expansion of monitoring parameters were needed to better assess changes of the interplay between climate and environmental or technical context of water supply.

## Supporting information

S1 Table P-values from permutation test for testing yearly periodicity in contamination time-series data. (DOCX)
