# Supplementary material for: The impact of rainfall on drinking water quality in Antananarivo, Madagascar
Source: PLoS One. 2020 Jun 15;15(6):e0218698. doi: 10.1371/journal.pone.0218698 (PMC7295214; doi:10.1371/journal.pone.0218698)
Supplement: S1 Table — (DOCX) [file pone.0218698.s001.docx]

**S1 Table. p values for testing significance of 12-months periodicity from permutation test on amplitude significance of time 12 periodograms.**

| **Periods** ^a)^ | **IE** ^b)^ | **EC** | **TC** | **SSRC** | **Rainfall** |
| --- | --- | --- | --- | --- | --- |
| 1985 (Jan) – 1989 (Oct) | NR | 0.105 | NR | 0.206 | NR |
| 1989 (Nov) – 2004 (Oct) | NR | 0.601 | NR | 0.519 | NR |
| 2004 (Nov) – 2012 (Feb) | NR | 0.390 | NR | 0.025 | NR |
| 2012 (Mar) – 2017 (Jul) | NR | 0.044 | NR | 0.012 | NR |
| Total periods | 0.010 ^c)^ | 0.3422 | 0.0007 | 0.0480 | 0.0000 |

a) year and (month) when breakpoint have occurred; b) Contamination markers, namely intestinal enterococci (IE), *Escherichia coli* (EC), total coliforms (TC) and spores of sulfite-reducing clostridia (SSRC); c)p-value from permutation test; if p-value < 0.05, there is an effect of month on the variable, i.e., contamination markers or rainfall. NR: not relevant.
